# Prostate Cancer Eligible for Radical Prostatectomy: Self-Esteem of Patients and Forms of Coping with Stress

**DOI:** 10.3390/ijerph19116928

**Published:** 2022-06-06

**Authors:** Edyta Skwirczyńska, Oskar Wróblewski, Karol Tejchman, Piotr Ostrowski, Natalia Serwin

**Affiliations:** 1 Department of History of Medicine and Medical Ethics, Pomeranian Medical University in Szczecin, 70-204 Szczecin, Poland; edyta.skwirczynska@pum.edu.pl; 2 Doctoral School, Pomeranian Medical University in Szczecin, 70-204 Szczecin, Poland; oskraw@gmail.com; 3 Department of General Surgery and Transplantation, Pomeranian Medical University in Szczecin, 70-204 Szczecin, Poland; karol.tejchman@pum.edu.pl (K.T.); piotr.ostrowski1997@gmail.com (P.O.); 4 Department of Laboratory Medicine, Pomeranian Medical University in Szczecin, 70-204 Szczecin, Poland

**Keywords:** self-esteem, prostate cancer, coping strategies, coping styles

## Abstract

The purpose of this study was to analyze the strategies and styles of coping with stress and self-esteem in patients diagnosed with prostate cancer. One hundred and five patients with prostate cancer participated in the study. Coping strategies were assessed with the Mini-Cope questionnaire, coping styles were assessed with the Coping Inventory for Stressful Situations, and self-esteem was assessed with the Rosenberg Self-Esteem Scale. Patients’ self-esteem and stress coping styles and strategies were analyzed using a Pearson correlation analysis. A stepwise linear regression analysis was performed to determine the predictors of self-esteem. The self-esteem level was positively related to the task-focused style (*r* = 0.228) and negatively related to the emotion-focused style (*r* = −0.329). The self-esteem level was significantly positively related to the strategies of active coping (*r* = 0.358), planning (*r* = 0.355), and seeking emotional support (*r* = 0.319) and was negatively related to self-blaming (*r* = −0.448) and to substance use (*r* = −0.301). The predictors of self-esteem level were: the strategies of self-blaming, planning, and the support-seeking dimension (F(3, 95) = 17.65; *p* < 0.001), explaining 33.8% of the variability in subjects’ self-esteem level. The moderating effect of age occurred in patients up to 65 years; it was statistically insignificant in patients older than 65 years. Replacement of the self-blame strategy and the emotion-focused style may lead to higher self-esteem of patients. The level of self-esteem can predict the strategies of self-blaming, planning, and the dimension of seeking support. For patients up to 65 years, psychological support should include reinforcement of adaptive forms of coping.

## 1. Introduction

A steady increase in cancer incidence has become a global problem [1]. One of the most common cancers among men is prostate cancer [2,3]. Factors considered important in prostate cancer disease development are mainly genetics, hormones, diet, and oxidative stress [3,4]. The most significant contribution to shaping health processes, which may affect the occurrence and course of neoplastic diseases, is attributed to lifestyle. One of its components that can positively or negatively affect health is the ability to cope with stress [5]. Self-esteem is considered an important factor in coping with cancer, which influences the experience of the illness itself, and its mediating role on psychosocial relationships is also important. It is also a personal resource that helps adapt to a new situation, such as the patient’s illness, and cope with its consequences [4].

The etiological diversity of cancer and the multiplicity of treatment forms are the main reason for the difficulty in indicating the relationship between stress and cancer processes. The identification of stress as the main factor responsible for neoplastic diseases is problematic due to the diverse etiology of tumors and associated diseases. The most significant difficulty in identifying stress as an important factor in cancer may be the time interval between the onset of stress and cancer development, as the accuracy of studies detecting pre-disease states is still insufficient. What remains undisputed is that stress modifies biological processes in both animals and humans and might cause disease onset and its further course. Patients are exposed to many postoperative complications that can significantly lower their self-esteem and lead to maladaptive forms of coping with stress. Moreover, self-esteem often decreases much earlier as a result of the diagnosed illness, caused by a deterioration in the physical appearance, a negative impact on personal relationships, reduced sexual function, false attributions related to cancer in the form of punishment, and depression [6]. It is estimated that 20 to 40% of patients suffer from psychological pain resulting in a negative impact on their overall health. This results in prolonged hospitalization, a decreased survival rate, and lowered self-esteem, following which patients are reluctant to undergo treatment [5,6,7]. Moreover, patients with cancer are more likely to develop depression than the general population, which, in addition to the drastically reduced quality of life of the patient and his family, may induce adverse immune changes, and these further affect neuroplasticity and neurotransmitter metabolism, and may cause gene modulation [8,9]. Most of these negative effects are strongly associated with chronic stress, which often accompanies diagnosis and treatment and also aggravates negative immune changes, thus promoting tumorigenesis and metastasis and reducing the effectiveness of anti-tumor therapies [10]. Furthermore, prostate cancer and radical prostatectomy significantly change the self-esteem, body image, and sense of masculinity in men with a diagnosis [11]. Besides being told they are seriously ill, men are faced with the fact that having a prostate removed can have a significant impact on their sex life, and the possible loss of sexual function is also difficult for them to discuss with doctors or even other men [11]. This often results in withdrawal from or a significant limitation of contacts and strongly influences their social life and often their families’ lives. 

With this in mind, it may be helpful for prostate cancer patients to identify styles and coping strategies associated with higher self-esteem. This may assist patients in intentionally choosing specific techniques and strategies that carry a positive psychological outcome in the form of higher self-esteem, which may significantly influence the course of treatment and recovery. Therefore, the aim of our study was to assess the psychological aspects during the treatment of prostate cancer in order to identify some of the predictors among styles and strategies of coping with stress in relation to patients’ self-esteem and further to evaluate the direction of the psychological help in such patients.

## 2. Materials and Methods

### 2.1. Study Design

The research was a cross-sectional study of self-esteem and styles and strategies for coping with stress in patients diagnosed with prostate cancer. Patients completed three questionnaires: the CISS questionnaire, Mini-Cope questionnaire, and the Rosenberg Self-Esteem Scale.

### 2.2. Participants

A total of 105 patients qualified for radical prostatectomy at the Independent Public Clinical Hospital No.2 of the Pomeranian Medical University in Szczecin, Department of Urology and Urological Oncology, and were invited to participate in the study from January 2018 to December 2020. Patients were qualified for radical prostatectomy by an oncology council consisting of an oncologist, urologist, radiotherapist, cancer coordinators, and a psycho-oncologist on the basis of a biopsy and imaging examination. The patients had a choice of treatment. Patients that qualified for radiotherapy were transferred to a specialized center cooperating with the clinic. Patients referred for chemotherapy underwent a different treatment regimen that depended on whether the method was used before or after tumor resection. For the cancer patients eligible for radical prostatectomy in this study, surgery was the first clinical contact in their treatment. Hence, the situation was completely new for them; the group was therefore considered relatively homogeneous in psychological terms.

The study was conducted by a psychologist while patients were waiting for surgery. After explaining the test procedure, the psychologist left the questionnaires and waited for their completion. Out of 112 questionnaires issued, 105 were returned.

The inclusion criterion for the study was the diagnosis of prostate cancer with a qualification for radical prostatectomy. The exclusion criteria were radiotherapy and hormone therapy, due to the specificity of such therapies and their possible complications; refusing to sign an informed consent form; and not returning completed questionnaires. 

All participants were informed about the purpose of the study and the possibility of withdrawing at any time and signed informed consent. Patients were excluded from the study if they did not sign the informed consent declaration of the respondent.

### 2.3. Procedure and Data Collection 

After signing the informed consent declaration, patients were invited to fill out the demographic questionnaire, the Coping Inventory for Stressful Situations (CISS) (Supplementary Materials, Questionnaire Q1), The Rosenberg Self-Esteem Scale (Supplementary Materials, Questionnaire Q2), and the Mini-COPE questionnaire (Supplementary Materials, Questionnaire Q3).

After being completed by the patients, the questionnaires were collected by the psychologist conducting the study. The demographic questionnaire included nine nominal independent variables, including age, place of residence (village, town with up to 100,000 inhabitants, or town with more than 100,000 inhabitants), education (primary, vocational, secondary, post-secondary, bachelor’s, or master’s-and-higher education), marital status (bachelor, married, divorced, or widower), children (yes/no and number of), satisfaction with the relationship with wife/partner (on a scale of 1–10), satisfaction with relationships with children (on a scale of 1–10), financial situation (on a scale of 1–10), and help from relatives and family (on a scale of 1–10). The scale’s reliability, depending on the age group, is 0.81 to 0.83. 

The Mini-Cope scale Polish adaptation of Ogińska-Bulik and Juczyński (2009) was used to examine coping strategies. This tool is used to measure dispositional coping. It is also known as Carver’s inventory and contains 28 statements included in 14 strategies for coping with stress (Supplementary Materials, Questionnaire Q3). The half reliability of the inventory was 0.86. The internal consistency for most of the scales was assessed at a satisfactory level [12].

The Coping Inventory for Stressful Situations (CISS), an adaptation of Strelau et.al., was used to examine coping styles [13]. The questionnaire contains 48 statements about events that are commonly considered stressful and specific patterns adopted when faced with these events. The scale identifies three main coping styles: task-focused, emotion-focused, and avoidance-focused. The avoidance-focused style can come in two forms: engaging in vicarious activities or seeking social contact. The questionnaire shows factor accuracy and a high internal consistency, measured using Cronbach’s alpha, in the range of 0.78–0.90.

The Rosenberg self-esteem scale Polish adaptation of Łaguna, Lachowicz-Tabaczek, and Dzwonkowska is a 10-level questionnaire used to measure the general level of self-esteem. The questionnaire contains 10 diagnostic statements and a four-level response scale assigned to them. The reliability of the scale varies depending on the age ranges, ranging from 0.81 to 0.83 [14,15].

### 2.4. Statistical Analysis

Data analysis was performed using IBM SPSS Statistics 25 with basic descriptive statistics analyses, including the Kolmogorov–Smirnov (K-S) test, Student’s *t*-tests for independent samples, correlation analyses with Pearson’s *r* coefficient, and stepwise linear regression analysis. The threshold of α = 0.05 was considered the significance level; however, test statistical probability scores of 0.05 < *p* < 0.1 were interpreted as significant at the level of a statistical trend.

## 3. Results

### 3.1. Demographic Data

The survey was completed by 105 of respondents. They were all Caucasian white men living in the West Pomeranian Voivodeship, mostly from smaller towns in the voivodeship (52.4%). Of all respondents, four were childless (3.8%). 

Detailed results of the demographic questionnaire are presented in Table 1 and Table 2.

### 3.2. Basic Descriptive Statistics of Measured Quantitative Variables

As shown in Table 3, distributions close to normal were recorded for the four styles of coping with stress (task, emotion, avoidance (*p* = 0.20), and distraction (*p* = 0.062)) and the two dimensions of coping strategies (active coping (*p* = 0.063) and avoidance (*p* = 0.076)). For the other variables studied, the distributions were different from the Gaussian distribution, as indicated by the statistically significant results of the K-S test. In this case, it is recommended to verify the skewness level. When the skewness value of the tested distributions is between −2 and +2, one can assume that they are not significantly asymmetric to the mean, which was observed for all of the studied variables. Therefore, it was decided that in this chapter, statistical analyses would be performed using parametric tests, of course, meeting their other assumptions.

### 3.3. Relationship between Self-Esteem and Coping Styles

A series of correlation analyses with Pearson’s *r* coefficient were performed. Two statistically significant relationships were observed. The level of self-esteem was positively related to the level of task-focused style and negatively associated with the emotion-focused style. This means that the level of self-esteem of the subjects increased with an increase in the task-focused coping style and decreased with an increase in the emotion-focused style. The strength of the former relationship was low, while the strength of the latter association was moderately high. One correlation at the level of the statistical trend was also noted: the scale of engaging in vicarious activities correlated negatively with the level of self-esteem of the subjects. However, the strength of this correlation was low. The remaining correlations appeared not to be statistically significant (Table 4).

### 3.4. Relationship between Self-Esteem and Coping Strategies

In the next step, we examined whether the level of self-esteem was related to the level of stress coping strategies. Another series of correlation analyses with Pearson’s *r* coefficient were performed. A series of statistically significant relationships were noted. The level of self-esteem was positively related to the level of active coping strategies, planning, seeking emotional support, seeking instrumental support, and the dimensions of active coping and seeking support and was negatively related to the level of substance abuse strategies, cessation of action, and blaming oneself and the dimension of helplessness. The strength of the associations between self-esteem and positive reappraisal, seeking instrumental support, and cessation strategies were low, while the remaining correlations were moderately strong. Two correlations at the level of a statistical trend were also noted. Discharge strategy and the dimension of avoidant behavior correlated negatively with the level of self-esteem. However, the strength of these correlations was low. The remaining correlations were not statistically significant (Table 5).

### 3.5. Styles and Strategies of Coping with Stress as Predictors of the Level of Self-Esteem

In the next step, a stepwise linear regression analysis was performed to verify which of the coping styles and strategies presented in the previous chapters were the best predictors of self-esteem level as the dependent variable. Thus, a stepwise linear regression analysis was performed. Three predictors were entered into the model: self-blame strategies, planning, and the support-seeking dimension (F(3, 95) = 17.65; *p* < 0.001). Together, these variables explained 33.8% of the variability in the subjects’ self-esteem levels. The remaining variables were not entered into the model because they did not significantly improve the level of explained variability. As can be seen in Table 6, the strategy of self-blaming proved to be the strongest predictor.

In summary, one should state that the level of self-esteem increased with a decrease in the level of the self-blaming strategy and an increase in the planning strategy and the seeking support dimension.

### 3.6. The Moderating Effect of Age on the Relationship between the Styles and Strategies of Coping with Stress and the Self-Esteem of Patients

As a final step, it was tested whether the age of the subjects was a statistically significant moderator of the relationships between any of the coping styles, strategies, and strategy dimensions and the self-esteem of patients. Thus, moderation analyses were performed using a series of regression analyzes (the “Process” macro) with age as the third dichotomous moderator variable. Age was considered as belonging to the age groups determined by Erikson’s stages, dividing the patients into two groups: up to 65 years and over 65 years.

When analyzing coping styles, there was a statistically significant moderation effect of age on the relationship between the task-focused style and self-esteem (*p* = 0.014, *R*^2^ = 0.048). The correlation between these variables was statistically significant in the group of people up to 65 years (*B* = 0.12; *SE* = 0.04; *t* = 2.86; *p* = 0.005), while in the group of people aged 65 and over, the relationship was not statistically significant (*B* = −0.01; *SE* = 0.07; *t* = −0.20; *p* = 0.846). Other moderating effects were not statistically significant concerning coping styles.

In coping strategies, there was a statistically significant moderation effect of age on the relationship between self-esteem and active coping (*p* = 0.006, *R*^2^ = 0.07), positive reappraisal (*p* = 0.018, *R*^2^ = 0.05), acceptance (*p* = 0.047, *R*^2^ = 0.04), and support seeking (*p* = 0.047, *R*^2^ = 0.04). The correlation between the active coping strategy and self-esteem was statistically significant in the group of people up to 65 years (*B* = 3.57; *SE* = 0.75; *t* = 4.79; *p* < 0.001), while in the group of people aged 65+, the relationship was not significant (*B* = 0.55; *SE* = 0.67; *t* = 0.73; *p* = 0.469). For positive reappraisal and self-esteem, the correlation was statistically significant in the group of people up to 65 years (*B* = 3.68; *SE* = 0.72; *t* = 3.68; *p* < 0.001) but not in the group of people aged 65+ (*B* = −0.26; *SE* = 0.96; *t* = −0.27; *p* = 0.788). A significant correlation between acceptance and self-esteem was also observed in patients up to 65 years (*B* = 2.10; *SE* = 0.92; *t* = 2.29; *p* = 0.024), while in the 65+ group the correlation was not significant (*B* = −0.46; *SE* = 0.87; *t* = −0.52; *p* = 0.604). The correlation between the support-seeking strategy and self-esteem in the group up to 65 was significant (*B* = 2.48; *SE* = 0.76; *t* = 3.27; *p* = 0.001), but it was not significant in the 65+ group (*B* = 0.28; *SE* = 0.79; *t* = 0.36; *p* = 0.721).

For the level of dimensions of coping strategies, there was a statistically significant moderation effect of age on the relationship between the self-esteem and the active coping (*p* = 0.015, *R*^2^ = 0.05) dimension and with the support-seeking dimension (*p* = 0.049, *R*^2^ = 0.04). The correlation for active coping dimension was statistically significant in the group of people up to 65 years of age (*B* = 4.25; *SE* = 0.86; *t* = 4.96; *p* < 0.001), while in the group of people aged 65+ the relationship was not statistically significant (*B* = 0.96; *SE* = 1.02; *t* = 0.95; *p* = 0.346). Moreover, similar observations were made for the correlation between self-esteem and the support-seeking dimension, which was significant in the group of people up to 65 years (*B* = 2.70; *SE* = 0.71; *t* = 3.80; *p* < 0.001), while in the group of people aged 65+, it was not statistically significant (*B* = 0.50; *SE* = 0.85; *t* = 0.59; *p* = 0.558).

## 4. Discussion

In the present study, we addressed the issue of self-esteem and coping strategies and styles used by patients in the context of prostate cancer, showing that the level of self-esteem depends on the choice of the form of coping with stress, mainly on self-blaming and planning strategies and the support seeking dimension. In patients diagnosed with cancer, it often relates to the patient’s ability to control the situation [16]. It has been indicated that the use of problem-focused strategies results in a higher quality of life and less frequent occurrences of depressive and anxiety symptoms in cancer patients. They are also better adjusted psychologically. At the same time, focusing on the emotional aspect is a factor affecting the incidence of anxiety and depressive symptoms [17]. 

Apart from the stress, self-esteem is also an important factor influencing both patients’ quality of life and coping with the disease. Eton, Lepore, and Helgeson showed that self-esteem and self-efficacy were significant predictors of quality of life [18]. They also demonstrated that psychological intervention was a significant moderator for psychological variables such as low self-esteem and low self-confidence. The present study on determining the relationships between self-esteem and stress coping strategies and styles in patients diagnosed with prostate cancer revealed several associations. First, the hypothesis regarding the associations between self-esteem and coping styles and strategies was confirmed. Patients who had higher self-esteem were more likely to use a task-focused style. When confronted with a stressor, patients with high self-esteem tend to analyze the problem in-depth, focusing on ways to solve it. The analysis also showed that self-esteem decreased in subjects using an emotion-focused style. This indicates that focusing on one’s own emotions, which in cancer patients, can occur as anger, resentment, and tension, negatively impacts self-esteem. The results indicate that patients with higher self-esteem use adaptive coping strategies in the form of active coping, planning, and seeking emotional and instrumental support. Ptacek et al. also showed that men with prostate cancer were most likely to use support-seeking and coping strategies. The researchers also indicated that younger men were more likely to focus on coping-based strategies when faced with a stressor [19]. A study by Roesch et al. on a group of men with prostate cancer also indicates the use of adaptive coping strategies to minimize the anxiety associated with the diagnosis [20]. The above studies seem to directly indicate the positive impact of adaptive coping strategies on stress. The present study also confirmed the hypothesis regarding the presence of self-esteem predictors in the form of self-blame, planning, and support-seeking strategies. The subjects using a strategy based on self-blaming showed a significant decrease in self-esteem. In contrast, using strategies based on planning and seeking support increased self-esteem. The final stage of the study examined whether there was a moderating effect of age on the relationships between coping styles and strategies and the subjects’ self-esteem. Age was shown to be a moderator of the relationship between the task-focused style and self-esteem. Age also moderated the associations between the active coping strategy, positive reappraisal, acceptance, emotional support, instrumental support, and self-esteem. A recurring trend was seen in all statistically significant strategies and the opinion-focused style. Among patients under 65 years, a more frequent use of coping strategies based on active coping, positive re-evaluation, acceptance, and seeking emotional and instrumental support resulted in higher levels of self-esteem. A similar trend was not seen in patients over 65 years. This can be explained by the fact that the compared groups differ in the relation of age to human developmental phases. People in the middle adulthood phase are confronted with the fear associated with the possibility of losing everything they have achieved and gained in life. People at this age are most burdened by the demands of family life, which is not only limited to their children but also to their parents, who are usually elderly. This is also the time of greatest social pressure, which causes even more tension. It can be reduced through creative activities or stagnation. Moreover, it seems that in patients under 65 years, the fact of having cancer has not negatively affected their activity. Most of them have families and children with whom they seek emotional support as a coping strategy. People in this age group also have the widest network of acquaintances resulting from their work and social life, which explains their search for instrumental support as a coping strategy. This may be because senior patients show decreases in both physical and social activity. According to Erikson, individuals in later adulthood take stock of their lives, which, depending on the outcome, results in life satisfaction or regret resulting from mistakes made. In patients older than 65 years, self-esteem is, perhaps, also less influenced by sexual function, as it is in young and middle-aged patients. The results are interesting because so far there has been no research in the literature on the moderating effect of age in the context of strategies and styles of coping with stress versus self-esteem. 

Basing on our study, we can conclude that patients under 65 years old belong to the group of actively functioning people, which translates into more frequent use of adaptive coping strategies and styles, resulting in their higher level of self-esteem. The study revealed many significant relationships between the variables. The overarching conclusion drawn from this study concerns the role of the psychologist in the patient treatment process. The aspect of psychological support, which has so far been underestimated, has increasingly emerged in the literature as significant in terms of faster and more effective treatment of patients. It is noteworthy that in similar studies patients themselves asked for psychological support and assistance. This is important because postoperative complications negatively impact the patient’s mental health and their relationship with their partner or spouse [21]. 

Another interesting and important finding of this study is the possibility of identifying the strategies and styles of coping with stress that can predict the formation of self-esteem in patients. The study showed that such strategies include self-blaming, planning, and seeking support, with the strongest variability observed around the strategy of self-blaming. There is a tendency for certain groups of patients to blame themselves for their illness, and sometimes patients see it as a kind of punishment. In prostate cancer, the stigma associated with prostate cancer as a self-inflicted illness is rather related to a diagnosis, not to a particular lifestyle or behavior, such as in, for example, lung, skin, or stomach cancer patients [22]. 

Working through these maladaptive statements in patients can increase their self-esteem, thus leading to better hospital outcomes and faster recovery. Moreover, working on adaptive strategies in planning the next steps after treatment and seeing support in the immediate family can contribute to higher self-esteem. It is also worth noting that the use of maladaptive coping strategies often leads to prolonged exposure to the stressor, and thus to its negative effects that can result in oxidative stress, the disruption of homeostasis, and even withdrawal from treatment.

A very important factor influencing the self-esteem of patients with prostate cancer is also society as well as some stereotypes or even the stigmatization of such people.

## 5. Conclusions

In prostate cancer patients, the level of self-esteem depends on their forms of coping with stress. Certain coping strategies and styles—the self-blaming strategy, planning strategy, and support seeking dimension—predict patients’ self-esteem. Our studies give reason to believe that an appropriate modification of maladaptive strategies into their adaptive forms can contribute to higher self-esteem in patients, and that, in turn, may lead to improving their well-being during the treatment process.

Our findings speak to the need for more extensive psychological care for patients, not only for crisis intervention but also for psychoeducational forms to support patient treatment. We provide preliminary evidence that modifying maladaptive forms of coping with stress might lead to higher self-esteem in patients, resulting in better well-being. These findings are important, not only for medical care but especially for patients and their families, who often have limited access to psychological care at the time of a significant crisis. The results indicate the necessity of holistic medical care linking medical, nursing, psychological, and physiotherapeutic care as a full range of assistance to the patient. It would be advisable to routinely introduce cancer patients to the possible ways of coping with stress, as many of them may not be aware of the impact of coping with stress on their daily functioning. Building an appropriate level of awareness will allow for the early psychoeducation of patients, contributing to the improvement of their self-esteem.

## 6. Limitations

The study has several limitations. First, it did not account for dynamics in the choice of styles and coping strategies for stress. Future longitudinal studies focusing on variability within the forms of coping with stress should be used. A second limitation of the present study was the relatively small study sample, which was due in part to the timing of the worldwide COVID-19 pandemic as well as the focus on only the selected cancer type of prostate cancer. It should also be noted that the correlations were not strong, as the mental state as well as its evaluation and definition are generally subjective feelings, even when accompanied by objective causes that may affect well-being. The obtained results indicate at a statistically significant level, however, that the issue is important from a psychological point of view and should be considered when assessing the patient’s condition and predicting the course of treatment, both before and after surgery.

The strengths of the study include the inclusion of a psychological factor in the treatment of patients. So far, they have focused on the social support of patients and the choice of therapy and course of treatment. Although the factors mentioned above are extremely important, the psychologist’s role in treatment deserves special attention.

## Figures and Tables

**Table 1:** Demographic data on age, number of children, and assessment of analyzed parameters of life satisfaction. M—mean; Me—median; SD—standard deviation; Min.—minimum; Max.—maximum.

	M	Me	SD	Min.	Max.
Age	62.8	54	5.43	48	75
No. of children	2.21	2.0	1.19	0	7
Satisfaction with contacts with children	9.26	10	1.10	5	10
Satisfaction with contacts with partner/wife	8.73	10	5.15	1	10
Help from family	7.58	9	2.95	1	10
Financial situation	7.03	7	1.91	1	10

**Table 2:** Demographic data on marital, educational, and residential status of patients.

		*N*	%
Place of residence	Village	18	17.1
	Town up to 100,000 inhabitants	55	52.4
	Town with more than 100,000 inhabitants	32	30.5
Marital status	Bachelor	1	1.0
	Married	92	87.6
	Divorced	8	7.6
	Widower	4	3.8
Education	Primary	8	7.6
	Vocational	34	32.4
	Secondary	33	31.4
	Post-secondary	2	1.9
	Bachelor	3	2.9
	Masters’ and higher	25	23.8

**Table 3:** Basic descriptive statistics for the quantitative variables.

		M	Me	SD	Sk.	Kurt.	Min.	Max.	D	*p*
CISS Stress coping styles	Task	53.63	54	10.51	−0.55	1.12	18	77	0.06	0.200
	Emotion	38.70	39	9.58	0.07	0	17	65	0.06	0.200
	Avoidance	41.47	42	9.35	−0.14	0.41	17	68	0.07	0.200
	Distraction	17.70	17	5.15	0.16	0.04	8	34	0.08	0.062
	Social Diversion	15.74	16	4.05	−0.59	0.52	4	24	0.10	0.015
Mini-COPE Stress coping strategies	Active coping	2.05	2	0.68	−0.42	−0.14	0	3	0.18	<0.001
	Planning	1.99	2	0.66	−0.07	−0.47	0.50	3.50	0.22	<0.001
	Positive reappraisal	1.68	1.50	0.64	−0.16	0.43	0	3	0.18	<0.001
	Acceptance	1.92	2	0.63	−0.10	−0.27	0.50	3	0.19	<0.001
	Sense of humor	0.97	1	0.63	0.33	−0.35	0	2.50	0.18	<0.001
	Turning to religion	1.07	1	0.95	0.43	−0.87	0	3	0.18	<0.001
	Seeking of emotional support	1.80	2	0.79	−0.23	−0.42	0	3	0.19	<0.001
	Seeking of instrumental support	1.72	2	0.71	−0.35	−0.11	0	3	0.19	<0.001
	Self-distraction	1.58	1.50	0.77	−0.10	−0.23	0	3.50	0.14	<0.001
	Denial	0.84	1	0.73	0.66	−0.24	0	2.50	0.18	<0.001
	Venting	1.10	1	0.64	−0.08	−0.94	0	2.50	0.18	<0.001
	Substance use	0.39	0	0.57	1.23	0.70	0	2.50	0.37	<0.001
	Behavioral disengagement	0.86	1	0.70	0.53	0.12	0	3	0.18	<0.001
	Self-blame	1.19	1	0.69	−0.06	−0.61	0	3	0.16	<0.001
Dimensions of strategies of coping with stress	Active coping	1.91	1.92	0.54	−0.02	−0.73	0.83	3	0.09	0.063
	Helplessness	0.81	0.83	0.49	0.39	−0.30	0	2.17	0.10	0.016
	Seeking of support	1.76	1.75	0.69	−0.29	−0.21	0	3	0.12	0.001
	Avoidance	1.17	1.17	0.52	0.03	−0.16	0	2.50	0.08	0.076
Rosenberg Score	Self-esteem	30	30	3.91	0.12	1.88	16	40	0.16	<0.001
	Age	63.76	64	6.09	−0.37	−0.49	48	75	0.11	0.003

**Table 4:** Relationship of self-esteem and coping styles; *r*—Pearson’s correlation coefficient. *p*—significance value.

Coping Style		Self-Esteem
Task	*r*	0.228
	*p*	0.020
Emotion	*r*	−0.329
	*p*	<0.001
Avoidance	*r*	−0.047
	*p*	0.634
Distraction	*r*	−0.184
	*p*	0.062
Social Diversion	*r*	0.150
	*p*	0.129

**Table 5:** Relationship of self-esteem and coping strategies; *r*—Pearson’s correlation coefficient. *p*—significance value.

Coping Strategy		Self-Esteem
Active coping	*r*	0.358
	*p*	<0.001
Planning	*r*	0.355
	*p*	<0.001
Positive reappraisal	*r*	0.265
	*p*	0.007
Acceptance	*r*	0.128
	*p*	0.207
Sense of humor	*r*	0.066
	*p*	0.519
Turning to religion	*r*	−0.048
	*p*	0.640
Seeking of emotional support	*r*	0.319
	*p*	0.001
Seeking of instrumental support	*r*	0.253
	*p*	0.010
Self-distraction	*r*	−0.050
	*p*	0.613
Denial	*r*	−0.142
	*p*	0.153
Venting	*r*	−0.174
	*p*	0.078
Substance use	*r*	−0.301
	*p*	0.002
Behavioral disengagement	*r*	−0.217
	*p*	0.028
Self-blame	*r*	−0.448
	*p*	<0.001
Active coping	*r*	0.397
	*p*	<0.001
Helplessness	*r*	−0.432
	*p*	<0.001
Seeking of support	*r*	0.313
	*p*	0.001
Avoidance	*r*	−0.164
	*p*	0.099

**Table 6:** Results of stepwise linear regression analysis for self-esteem level as the dependent variable.

	*B*	*SE*	Beta	*t*	*p*
	27.85	1.21		22.95	<0.001
Self-blame	−2.44	0.46	−0.44	−5.30	<0.001
Planning	1.56	0.54	0.27	2.91	0.005
Seeking of support	1.13	0.52	0.20	2.18	0.032

## Data Availability

Not applicable.

## Supplementary Materials

The following supporting information can be downloaded at: https://www.mdpi.com/article/10.3390/ijerph19116928/s1, Questionnaire Q1. the Coping Inventory for Stressful Situations (CISS); Questionnaire Q2. The Rosenberg Self-Esteem Scale; Questionnaire Q3. The Mini-Cope scale.
