# Supplementary material for: Prostate Cancer Eligible for Radical Prostatectomy: Self-Esteem of Patients and Forms of Coping with Stress
Source: Int J Environ Res Public Health. 2022 Jun 6;19(11):6928. doi: 10.3390/ijerph19116928 (PMC9180423; doi:10.3390/ijerph19116928)
Supplement: Supplementary file 1 [file ijerph-19-06928-s001.zip › ijerph-1647851-supplementary.pdf]

Supplementary Materials

Questionnaire Q1. the Coping Inventory for Stressful Situations (CISS).

The Coping Inventory for Stressful Situations

Norman S. Endler & James D. A. Parker

Name and surname …………………………… Age……………….

Gender ………………………

Education ………………………. Occupation …………………. Date ………………

INSTRUCTIONS: the following sentences describe different people's reactions to difficult, unpleasant, stressful situations. Circle one of the numbers from 1 to 5 next to each statement. In this way, determine how much you engage in these activities when you find yourself in a difficult, unpleasant, stressful situation.

1 – never
2 – bardzo rzadko
3 – czasami
4 – often
5 - very often

1. Schedule my time better 1 2 3 4 5
2. Focus on the problem and see how I can solve it 1 2 3 4 5
3. Think about the good times in the past 1 2 3 4 5
4. Try to be with other people 1 2 3 4 5
5. Blame myself for procrastinating 1 2 3 4 5
6. Do what I think is best 1 2 3 4 5
7. Preoccupied with aches and pain 1 2 3 4 5
8. Blame myself for having gotten into this situation 1 2 3 4 5
9. Window shopping 1 2 3 4 5
10. Outline my priorities 1 2 3 4 5
11. Try to sleep 1 2 3 4 5
12. Treat myself to a favorite food or snack 1 2 3 4 5
13. Feel anxious about not being able to cope 1 2 3 4 5
14. Become very tense 1 2 3 4 5
15. Think about how I have solved similar problems 1 2 3 4 5
16. Tell myself "it's not really happening" 1 2 3 4 5
17. Blame myself for being too emotional 1 2 3 4 5
18. Go out for a snack or ameal 1 2 3 4 5
19. Become very upset 1 2 3 4 5
20. Buy myself something 1 2 3 4 5
21. Determine a course of action and follow it 1 2 3 4 5
22. Blame myself for not knowing what to do 1 2 3 4 5
23. Go to a party 1 2 3 4 5

24. Work to understand the situation... 1 2 3 4 5
25. Freeze and don't know what to do 1 2 3 4 5
26. Take corrective action immediately 1 2 3 4 5
27. Think about the event and learn from my mistakes 1 2 3 4 5
28. Wish that I could change what happened, or how I felt 1 2 3 4 5
29. Visit a friend 1 2 3 4 5
30. Worry about what I should do... 1 2 3 4 5
31. Spend time with a special person.. 1 2 3 4 5
32. Go for a walk... 1 2 3 4 5
33. Tell myself "it will never happen again".. 1 2 3 4 5
34. Focus on my general inadequacies 1 2 3 4 5
35. Talk to someone whose advice I value 1 2 3 4 5
36. Analyze the problem before reacting.. 1 2 3 4 5
37. Phone a friend 1 2 3 4 5
38. Get angry... 1 2 3 4 5
39. Adjust my priorities... 1 2 3 4 5
40. See a movie... 1 2 3 4 5
41. Get control of the situation 1 2 3 4 5
42. Make an extra effort to get things done 1 2 3 4 5
43. Come up with several different solutions 1 2 3 4 5
44. Take time off and get away from the situation 1 2 3 4 5
45. Take it out on others... 1 2 3 4 5
46. Use the situation to prove my ability.. 1 2 3 4 5
47. Try to be organized for the next time like this 1 2 3 4 5
48. Watch TV 1 2 3 4 5

Questionnaire Q2. The Rosenberg Self-Esteem Scale

The Rosenberg Self-Esteem Scale

Below is a list of statements dealing with your general feelings about yourself. Please indicate how strongly you agree or disagree with each statement by circling one of the four possible responses. Try to identify what you really think. Only honest answers count.

1. On the whole, I am satisfied with myself.

Strongly Agree Agree Disagree Strongly Disagree

2. At times I think I am no good at all.

Strongly Agree Agree Disagree Strongly Disagree

3. I feel that I have a number of good qualities.

Strongly Agree Agree Disagree Strongly Disagree

4. I am able to do things as well as most other people.

Strongly Agree Agree Disagree Strongly Disagree

5. I feel I do not have much to be proud of.

Strongly Agree Agree Disagree Strongly Disagree

6. I certainly feel useless at times.

Strongly Agree Agree Disagree Strongly Disagree

7. I feel that I'm a person of worth, at least on an equal plane with others.

Strongly Agree Agree Disagree Strongly Disagree

8. I wish I could have more respect for myself.

Strongly Agree Agree Disagree Strongly Disagree

9. All in all, I am inclined to feel that I am a failure.

Strongly Agree Agree Disagree Strongly Disagree

10. I take a positive attitude toward myself.

Strongly Agree Agree Disagree Strongly Disagree

Reference:

Rosenberg, M. (1965). Society and the adolescent self-image. Princeton, NJ: Princeton University Press.

Questionnaire Q3. The Mini-Cope scale

Charles Carver

Mini-COPE

In each line, mark one answer that suits you best by circling the appropriate number (0–1–2–3). Do not omit any statement.
The numbers mean:

0 = Not at all
1 = Little bit
2 = Medium amount
3 = Doing a lot

1. I've been turning to work or other activities to take my mind off things...........0 1 2 3
2. I’ve been concentrating my efforts on doing something about the situation I’m in. 0 1 2 3
3. I've been saying to myself "this isn't real." 0 1 2 3
4. I've been using addictive behaviors or substances to make myself feel better. 0 1 2 3
5. I've been getting emotional support from others. 0 1 2 3
6. I've been giving up trying to deal with it. 0 1 2 3
7. I've been taking action to try to make the situation better. 0 1 2 3
8. I've been refusing to believe that it has happened. 0 1 2 3
9. I've been saying things to let my unpleasant feelings escape. 0 1 2 3
10. I’ve been getting help and advice from other people. 0 1 2 3
11.I've been using alcohol or other drugs to help me get through it. 0 1 2 3
12. I've been trying to see it in a different light, to make it seem more positive. 0 1 2 3
13. I’ve been criticizing myself. 0 1 2 3
14. I've been trying to come up with a strategy about what to do. 0 1 2 3
15. I've been getting comfort and understanding from someone. 0 1 2 3
16. I've been giving up the attempt to cope. 0 1 2 3
17. I've been looking for something good in what is happening. 0 1 2 3
18. I've been making jokes about it. 0 1 2 3
19. I've been doing something to think about it less, such as going to movies, watching TV, reading, daydreaming, sleeping, or shopping. 0 1 2 3
20. I've been accepting the reality of the fact that it has happened. 0 1 2 3
21. I've been expressing my negative feelings. 0 1 2 3
22. I've been trying to find comfort in my religion or spiritual beliefs. 0 1 2 3
23. I’ve been trying to get advice or help from other people about what to do. 24. I've been learning to live with it. 0 1 2 3
25. I've been thinking hard about what steps to take. 0 1 2 3
26. I’ve been blaming myself for things that happened. 0 1 2 3
27. I've been praying or meditating. 0 1 2 3
28. I've been making fun of the situation. 0 1 2 3
